# Association between school commuting and adolescent mental health: Insights from a longitudinal study amid the pandemic

**DOI:** 10.1371/journal.pmen.0000159

**Published:** 2024-10-30

**Authors:** Gesse Ferreira Lima, Paulo Nascimento Neto, Adriano Akira Hino, Fabio Duarte

**Affiliations:** 1 Graduate Program in Urban Management, Pontifícia Universidade Católica do Paraná, Curitiba, Brazil; 2 Graduate Program in Health Sciences, Pontifícia Universidade Católica do Paraná, Curitiba, Brazil; 3 Department of Urban Studies and Planning, Massachusetts Institute of Technology, Cambridge, Massachusetts, United States of America; Universidad de Huelva Facultad de Educacion, SPAIN

## Abstract

This study investigates the relationship between transportation modes and adolescent mental health, employing a natural experiment design to longitudinally track Brazilian high-school students amid the COVID-19 pandemic disruptions. We surveyed 213 students in the city of Curitiba during and after the COVID-19 lockdown period using the Depression, Anxiety, and Stress Scale (DASS-21) combined with a socioeconomic questionnaire. To analyze changes in DASS between these two times we used the paired-sample T-test, exploring association with school commuting modes through binomial logistic regression models. The use of public transportation emerges as a significant factor associated with a threefold odds of increasing depression levels among students (OR = 3.08). This likelihood nearly quadrupled (OR = 3.81) when considering students living 5 km or more from school. These findings unveil public transportation and home-school distance as critical factors in the development of depression among adolescents. For example, individuals who commute via individual motorized transportation demonstrate a markedly lower likelihood of experiencing depression, pointing to broader societal and environmental factors such as family income and access to opportunities. Despite appearing contradictory to the commonly understood impacts of car use on mental health, this observation may unveil overlapping layers of spatial inequality and underscore the nuanced dynamics between transportation modes and adolescent mental well-being.

## Introduction

Over the past two decades, the influence of the built environment on physical and mental health has become a focal point in the realms of public health and urban studies. A comprehensive research agenda emerged in the early 2000s intending to identify factors that either mitigate or amplify the health implications of the built environment [1–4]. Since then, subsequent research has significantly contributed to elucidating how urban amenities [5], green spaces [6,7], housing conditions [8,9], and segregation patterns [10] impacts on individuals’ health and "healthy cities" [11].

In transportation research, a substantial body of literature consistently indicates a relationship between commuting duration to work and its significant impact on the mental well-being of adults [12–17]. Similarly, some studies have proposed that extended commuting times and exposure to high-traffic environments may have detrimental effects on the academic performance of children and adolescents [18,19], potentially leading to sleep disturbances [20]. Exogenous factors in the educational process, including the characteristics of commuting to school, influence students’ academic results [21].

In a context where approximately half of all enduring mental health conditions manifest before the age of 14, there exists a critical need to further investigate the nexus between urban environments and the psychological well-being of adolescents [7]. It is estimated that approximately one in three adolescents (34%) is at risk of developing clinical depression [22]. Among U.S. adolescents, data from National Comorbidity Survey Adolescent Supplement estimates a prevalence of 32% of any anxiety disorder in 2010. Furthermore, trends analysis has shown an increase in this prevalence from 34% in 2012 to 44% in 2018 [23].

However, while global estimations underscore the prevalence of depression and anxiety within this demographic cohort [24,25], research suggests that opportunities for active commuting in high-quality environments have a positive impact on the health level of young individuals [26–29].

Nonetheless, a more precise comprehension of this impact on the psychological well-being of children and adolescents remains an important area of investigation [30–32]. Much of the existing research only scratches the surface of the topic, often focusing solely on the effects of car usage for daily commutes and its implications for the environment and physical health (e.g., [33–35]). Concurrently, active commuting is believed to foster healthy and sustainable habits, potentially reducing perceived stress [36]. Furthermore, a body of literature suggests the positive impact of bus commuting among adolescents aged 12 to 18, fostering autonomy and self-regulation, facilitating social interaction, and encouraging physical activity, particularly during the walk to the bus stop [37].

Especially in Latin America, research focusing on the urban mobility of students through evidence-based analyses remains limited, primarily due to the scarcity of comprehensive data on population movements based on motivation, further compounded by the absence of dedicated research on school commuting [38]. In this context, this article aims to deepen the understanding of the relationship between school commuting and the mental health of young students.

The closure of schools and suspension of commuting activities due to the COVID-19 pandemic offered a unique setting for a natural experiment, enabling the longitudinal tracking of students’ mental well-being. Interrupting in-person classes disrupted students’ commuting routines in Brazil between March 2020 and February 2022, when in-person classes resumed, and transportation modes were reinstated.

This study investigates the relationship between transportation modes and adolescent mental health. Through a longitudinal study, we tracked a cohort of Brazilian high-school students from the onset of the pandemic restrictions (online classes, no commuting) to understand the impact of resuming transportation modes once the restrictions were lifted on depression, anxiety, and stress levels.

## Materials and methods

### Study design

The study employs a natural experiment design, tracking a cohort of participants over time. Natural experiments in social sciences resemble observational studies, bolstering empirical inferences [39]. While experimental studies hold merit, they face challenges in assessing before-and-after scenarios, thus limiting their findings [40]. Leveraging naturally occurring opportunities, this study evaluates the impact of transportation-related variables on mental health. Benefiting from a unique data collection window spanning the suspension of in-person classes due to the COVID-19 pandemic, in March 2020, when classes moved to an online mode, to the resumption of in-person classes, in February 2022. Thus, the investigation assesses participants twice: during remote learning without commuting and upon the subsequent return to daily home-to-school commutes. This two-phase approach offers insights into the potential influence of transportation modes on youth mental health.

### Study participants

The research was conducted in Curitiba, Brazil, renowned for its urban and transportation planning and one of Brazil’s major metropolitan areas. According to the Brazilian School Census [41], 59,882 high-school students were enrolled in public schools in Curitiba in 2020. For this paper, schools were selected based on their nature (we only selected public schools) and the institutional acceptance in participating in this study, comprising a total population of 3,500 students.

In the study’s first phase (online classes during the COVID-19 pandemic), we ran the experiment with 237 high-school students, between November 18, 2020, and December 18, 2020. The participants were selected randomly, and the sample represents those who accepted to participate in the study. In the second phase (in-person classes, post-pandemic, between March 22, 2022, and April 29, 2022), 213 out of the original 237 respondents (91.1%) participated, forming the sample analyzed in this study. The sample size provides a 95% confidence with a 6.5% margin of error. Additionally, considering a conservative estimated effect size (0.25) and three groups of transportation modes, the sample size presents a power of 91% (F = 3.03, df = 210).

Data collection was preceded by obtaining informed guardians’ consent for students under 18 years. The Research Ethics Committee of the State Education Department approved the research following Resolution 406/2018 GS-SEED. Furthermore, the University Research Ethics Committee approved it under process number CAAE 51345321.6.0000.0020. Ethical considerations were observed to ensure participants’ integrity and compliance with Brazilian law.

### Measurements

To explore the impact of transportation on students’ mental health, we utilized the DASS-21. The DASS is an instrument developed in 1995 to assess symptoms of depression and anxiety [42]. Stress was identified as a third factor during its development and data analysis. The scale was conceptualized as a 42-item instrument, comprising three subscales, each with 14 items representing symptoms experienced during the previous week. In 1998, a reduced version of the instrument (DASS-21) was tested, showing similar psychometric characteristics, with Cronbach’s alpha for depression, anxiety, and stress being 0.94, 0.87, and 0.91, respectively [43]. The Portuguese version of the DASS-21 has demonstrated suitable levels of internal consistency, ranging between 0.83 and 0.90, and confirmatory validity of the original three-factor model with dimensions of depression, anxiety, and stress [44]. The version utilized here comprises three subscales: depression, anxiety, and stress, each containing seven items assessing the respondent’s emotional state [45–48]. The DASS-21 questionnaire assesses how the individual felt during the week preceding their response, using a four-point Likert scale (0, not applicable, to 3, very applicable). The outcome of our study was the variation (ΔS) between time 1 (pandemic period) and time 2 (post-pandemic period) of the depression, anxiety, and stress subscales, which were dichotomized to reflect those who remained unchanged or decreased (Δ≤0), coded as "0", compared to those who increased, coded as "1".

The independent variable, mode of transportation to school, was self-reported based on the socioeconomic questionnaire. The answers were categorized into active commuting (walking or bicycling), individual motorized (motorcycling or by car), or public transportation. Sex, family income, school, and regular physical activity were self-reported and analyzed as potential confounding variables in the adjusted model.

### Statistical analysis

We used mean and standard deviation to describe quantitative variables and absolute and relative frequencies to describe the qualitative variable. To analyze changes in depression, anxiety, and stress between time 1 and time 2, the paired-sample T-test was used. The association between transportation mode and increase of depression, anxiety, and stress was tested using binomial logistic regression models. Crude and adjusted models were employed, considering the relationship between each transportation mode and all others (e.g., active commuting *versus* public transportation plus individual motorized). The variables sex, family income, school, and regular physical activity were included in the adjusted model as potential confounders.

The results were stratified by the respondents’ travel distance. A substantial body of literature has explored the relationship between active commuting and a specific range of distances from school, although the metrics remain non-consensual among authors [49–54]. This research adopts a 5 km threshold, equivalent to more than 60% of urban trips worldwide [55,56], commonly considered for active commuting in Brazil (e.g., [57]).

Given the absence of research on the association between mental health and school commuting in Latin America, we adopted a significance level of 10% (p-value ≤ 0.10). This decision is justified by the study’s exploratory nature and the phenomenon’s complexity under investigation, considering the high statistical power (> 90%) and the potential practical implications. This threshold facilitates a more comprehensive exploration of the association between transportation modes and adolescent mental health. Additionally, strictly adhering to a 5% significance level in studies with small sample sizes may increase the likelihood of type II errors, where true effects remain undetected. We can mitigate the risk of overlooking potentially significant relationships by opting for a slightly higher significance level.

## Results

The study participants (n = 213), aged between 14 and 18 years, were predominantly female (66%) and primarily from low-income families (76%). Public transportation was the most common mode of commuting (62%), followed by private motorized modes (22%), and active commuting (16%). Most youths reported engaging in regular physical activities (65%). Analysis of the data revealed a general decrease in the scale of depression (from 1.49 to 1.38), anxiety (1.52 to 1.38), and stress (1.43 to 1.31) levels following the resumption of in-person classes (Time 2; see Table 1).

**Table 1:** Results in pandemic (T1) and post-pandemic period (T2).

	Participants		Depression						Anxiety						Stress					
			Time 1		Time 2		Paired-sample T-test		Time 1		Time 2		Paired-sample T-test		Time 1		Time 2		Paired-sample T-test	
	n	%	Mean	SD	Mean	SD	*t*	Cohen’s d	Mean	SD	Mean	SD	*t*	Cohen’s d	Mean	SD	Mean	SD	*t*	Cohen’s d
**Total**	213	100%	1.49	0.82	1.38	0.72	t = 5.57*	0.38	1.52	0.75	1.38	0.68	*t* = 7.81*	0.54	1.43	0.82	1.31	0.69	*t* = 5.12*	0.35
**Sex**																				
Female	141	66%	1.67	0.78	1.54	0.67	t = 4.85*	0.41	1.66	0.71	1.52	0.64	*t* = 5.84*	0.49	1.58	0.81	1.43	0.68	*t* = 4.57*	0.38
Male	72	44%	1.15	0.80	1.09	0.73	t = 3.05*	0.36	1.23	0.74	1.11	0.68	*t* = 5.87*	0.70	1.12	0.77	1.06	0.65	*t* = 2.39*	0.28
**Transportation modes**																				
Public Transportation	132	62%	1.49	0.82	1.41	0.72	t = 3.48*	0.30	1.55	0.75	1.42	0.67	*t* = 5.59*	0.49	1.43	0.84	1.33	0.71	*t* = 3.52*	0.31
Activity Commuting	34	16%	1.36	0.83	1.21	0.70	t = 2.60*	0.45	1.38	0.75	1.16	0.64	*t* = 3.97*	0.68	1.31	0.77	1.15	0.59	*t* = 2.11*	0.36
Individual Motorized	47	22%	1.60	0.80	1.44	0.73	t = 3.78*	0.55	1.54	0.76	1.43	0.71	*t* = 4.02*	0.59	1.48	0.80	1.35	0.69	*t* = 3.57*	0.52
**Family Income** **																				
< 4 m.w.	162	76%	1.54	0.84	1.42	0.75	t = 5.34*	0.42	1.53	0.77	1.41	0.70	*t* = 6.27*	0.49	1.45	0.84	1.33	0.72	*t* = 4.70*	0.37
> 4 m.w.	51	24%	1.35	0.72	1.28	0.60	t = 1.86	0.26	1.48	0.69	1.31	0.62	*t* = 4.76*	0.67	1.33	0.74	1.24	0.61	*t* = 2.06*	0.29
**Physical Activity**																				
Yes	138	65%	1.39	0.84	1.28	0.76	t = 4.47*	0.38	1.44	0.77	1.32	0.71	*t* = 6.93*	0.59	1.34	0.84	1.23	0.71	*t* = 4.04*	0.34
No	75	35%	1.70	0.74	1.57	0.61	t = 3.34*	0.39	1.66	0.70	1.50	0.60	*t* = 4.24*	0.49	1.57	0.76	1.44	0.64	*t* = 3.14*	0.36
**School**																				
School A	52	24%	1.45	0.87	1.36	0.77	t = 2.50*	0.35	1.52	0.84	1.40	0.74	*t* = 3.16*	0.44	1.43	0.85	1.35	0.70	*t* = 1.79	0.25
School B	161	76%	1.51	0.81	1.39	0.70	t = 4.96*	0.39	1.52	0.72	1.38	0.66	*t* = 7.21*	0.57	1.42	0.81	1.29	0.69	*t* = 4.87*	0.38

Source: The authors. Note: * represents statistically significant at p-value ≤ 0.10. / ** Minimum wage equivalent to US$ 254 (reference: Brazil—2022).

We conducted paired samples t-tests to examine whether variations in depression, anxiety, and stress levels between times 1 (pandemic period) and 2 (post-pandemic period) were statistically significant.

Results of the variations in depression, anxiety, and stress levels between times 1 and 2 are presented for the entire sample and stratified by sex, transportation modes, family income, physical activity, and school (see Table 1). It is noteworthy that, with few exceptions (such as depression among higher-income participants and stress among students from School A), the subgroups also exhibit statistically significant differences in the measured levels at a significance level of 10%, confirming the noted improvement in mental health conditions after the resumption of classes. Furthermore, the data suggests variations in the mental health level of respondents grouped by transportation mode–notably, the active commuting group demonstrates generally lower averages of depression, anxiety, and stress during the pandemic period and after the resumption of in-person classes.

The associations between students’ modes of transportation and the increase in depression, anxiety and stress levels are presented on Table 2. Notably, school commuting via public transportation is associated with a threefold increase in the odds of anxiety level (OR = 3.08, p < 0.10) when compared to other transportation modes. Conversely, utilizing individual motorized modes of transportation suggests an 82% reduction in the odds of increasing depression levels (OR = 0.18, p < 0.10). No statistically significant associations were observed for stress and anxiety levels across any transportation mode. Finally, active commuting did not influence depression levels.

**Table 2:** Binary logistic regression model.

Entire sample (n = 213)						
	Increase in Depression Level OR [95%C.I.]		Increase in Anxiety Level OR [95%C.I.]		Increase in Stress Level OR [95%C.I.]	
	Crude	Adjusted	Crude	Adjusted	Crude	Adjusted
Active commuting (ref: 1)	1.06 [0.38–3.00]	-	0.60 [0.13–2.71]	-	1.46 [0.58–3.69]	-
Public transportation (ref: 2)	**2.23 [0.91–5.47]** *	**3.08 [1.19–7.98]** *	1.80 [0.62–5.21]	-	1.15 [0.54–2.47]	-
Individual motorized (ref: 3)	**0.22 [0.05–0.96]** *	**0.18 [0.04–0.82]** *	0.64 [0.18–2.29]	-	0.56 [020–1.54]	-

Source: The authors. Note: * represents statistically significant at p-value ≤ 0.10 / Confounding variables: Sex, income, school, and physical activity. / ref:1 = public transportation plus individual motorized / ref:2 = active commuting plus individual motorized / ref: 3 = active commuting plus public transportation.

In this context, when examining the association between depression, anxiety, and stress with transportation modes within the threshold of 5 km (a contextual limit for active commuting in Brazil), the analysis reveals no meaningful association between transportation modes and the increase in mental health issues (Table 3). Nevertheless, when considering students living 5 km or more from their school, there is a fourfold higher odds (OR 3.81, p < 0.10) of these students experiencing increased depression levels when using public transportation compared to those who use other modes of transportation. Active commuting was not statistically associated with mental health outcomes in both commuting thresholds.

**Table 3:** Binomial Logistic Models considering distance threshold.

**Commuting distance < 5km (N = 73)**						
	Increase in Depression Level OR [95%C.I.]		Increase in Anxiety Level OR [95%C.I.]		Increase in Stress Level OR [95%C.I.]	
	Crude	Adjusted	Crude	Adjusted	Crude	Adjusted
Active commuting (ref: 1)	3.30 [0.67–16.16]	-	0.71 [0.07–7.24]	-	1.89 [0.46–7.84]	-
Public transportation (ref: 2)	0.80 [0.17–3.84]	-	1.09 [0.15–8.19]	-	0.85 [0.21–3.46]	-
Individual motorized (ref: 3)	0.00 [0.00 –Inf]	-	1.31 [0.13–13.57]	-	0.46 [0.05–3.88]	-
**Commuting distance ≥ 5km (N = 140)**						
	Increase in Depression Level OR [95%C.I.]		Increase in Anxiety Level OR [95%C.I.]		Increase in Stress Level OR [95%C.I.]	
	Crude	Adjusted	Crude	Adjusted	Crude	Adjusted
Active commuting (ref: 1)	0.49 [0.06–3.99]	-	0.82 [0.10–6.90]	-	1.82 [0.45–7.43	-
Public transportation (ref: 2)	**3.46 [0.97–12.36]** *	**3.81 [1.00–14.52]** *	1.88 [0.50–7.04]	-	1.17 [0.45–3.05]	-
Individual motorized (ref: 3)	0.28 [0.06–1.25]	-	0.49 [0.10–2.28]	-	0.59 [0.19–1.87]	-

Source: The authors. Note: * represents statistically significant at p-value ≤ 0.10 / Confounding variables: Sex, income, school, and physical activity./ ref:1 = public transportation plus individual motorized / ref:2 = active commuting plus individual motorized / ref: 3 = active commuting plus public transportation.

## Discussion

The findings underscore the nuanced impact of transportation modes on adolescent mental health, particularly concerning depression levels (Table 2). The active commuting group consistently demonstrates lower averages of stress, anxiety, and depression both during the pandemic and after the resumption of in-person classes. These results are in line with a consistent body of research, including studies in Asia [58], North America [59], and Oceania [60], emphasizing the role of active school commuting in adolescents’ mental health [52,61,62].

Moreover, those who use public transportation for commuting demonstrate a significantly increased risk of experiencing depression, underscoring the imperative for tailored interventions and policy initiatives to protect the mental health of this cohort. These findings gain significance considering the prevalent focus of public policies and media on commuting to workplaces, often neglecting the specific needs of young individuals, as evidenced recently [63].

Our research also explored the role of distance thresholds in elucidating this intricate relationship, suggesting that accessibility levels and transportation modes influence adolescents’ mental health (Table 3). Distances greater than 5 km are associated with a notable increase in depression likelihood among adolescents. To some extent, as a proxy for long travels, this pattern suggests a relationship between mental health, the level of service, and the time taken on public transport, echoing contemporary observations [64,65].

However, caution is warranted when interpreting these results. While prior studies suggest positive impacts of independent commuting and active transportation modes on psychological development and overall satisfaction, our findings did not establish statistically significant associations between transportation modes and stress and anxiety levels–only with depression. On the other hand, in terms of internal validity, we evaluated the outcome using a well-established instrument that has shown high levels of validity and reliability [66]. The association analyses were conducted considering all available potential confounding variables. Data collection was carefully conducted to minimize dropout, achieving a follow-up rate of more than 90% among the students. Regarding external validity, we employed a natural experimental design, which allowed us to observe changes over time without any interference with the study variables.

In this regard, individuals who commute via individual motorized transportation demonstrate a markedly lower likelihood of experiencing depression, pointing to broader societal and environmental factors such as family income and access to opportunities. Despite appearing contradictory to the commonly understood impacts of car use on mental health, this observation may unveil overlapping layers of spatial inequality. It is relevant to recognize that the research was conducted in Brazil, a country characterized by pronounced socio-spatial inequality, reflected in disparities in vehicle ownership and the demographics of public-school students, who predominantly come from lower-income families. While a definitively causal relationship could not be established within the scope of this study, the overall analysis indicates that adolescents from families with higher incomes tend to exhibit lower levels of stress and depression (Table 1), suggesting a potential link between individual motorized commuting and better socioeconomic conditions, which may mitigate exposure to stressors. This conclusion aligns with observations in Asia [67], where the use of active commuting decreases as the average income increases.

Furthermore, it is essential to contextualize our findings within the specific urban landscape of Curitiba, a city globally recognized for its well-established public transportation system [68,69]. The high quality of Curitiba’s Bus Rapid Transit (BRT) corridors and feeder buses may influence the observed association between transportation modes and anxiety or stress levels, highlighting the need for broader sampling in future research to mitigate potential biases and further elucidate the complex interplay between transportation infrastructure and adolescent mental health.

## Conclusion

The present research examined the relationship between transportation modes and adolescent mental health, focusing on depression, anxiety, and stress. Through a longitudinal natural experiment, we tracked a cohort of Brazilian high-school students over a two-year period, spanning from the pandemic-induced restrictions (online classes, no commuting) to the resumption of commuting once the restrictions were lifted. The findings reveal a heightened risk of depression among students who rely on public transportation for school commutes, particularly when travel distances exceed 5 km. These results underscore that the transportation modality *per se* cannot serve as a singular explanatory factor, requiring contextualization within broader spatial features. Moreover, the active commuting group demonstrates generally lower averages of depression, anxiety, and stress, whether during the pandemic period or after the resumption of in-person classes.

The findings also challenge oversimplified assumptions about the inherent risks associated with different transportation modes, as demonstrated by the case of individual motorized commuting. Instead, they underscore the importance of considering the specific conditions under which each modality operates within the urban landscape–in our case, framed by the studied case, the city of Curitiba, Brazil. This points to the relevance of spatial planning and design decisions in promoting mental health among adolescents. In this context, despite the strengths of the longitudinal design, the study’s focus on a specific cohort may limit the generalizability to other populations or geographic contexts. Additionally, unmeasured confounding variables may influence the observed associations between transportation modes and mental health outcomes. Expanding upon this research, future studies could further explore these complex dynamics to inform targeted urban policies and spatial interventions within the urban environment.

## Supporting information

S1 Checklist (DOCX)

S1 Data (XLSX)
